# ﻿Two new species of *Orchestina* Simon, 1882 (Araneae, Oonopidae) from Cangshan Mountain, Yunnan, China

**DOI:** 10.3897/zookeys.1195.117666

**Published:** 2024-03-15

**Authors:** Xiaohan Wang, Dongju Bian, Yanfeng Tong, Zizhong Yang

**Affiliations:** 1 College of Life Science, Shenyang Normal University, Shenyang 110034, Liaoning, China Shenyang Normal University Shenyang China; 2 Key Laboratory of Forest Ecology and Management, Institute of Applied Ecology, Chinese Academy of Sciences, Shenyang 110016, China Institute of Applied Ecology, Chinese Academy of Sciences Shenyang China; 3 National-Local Joint Engineering Research Center of Entomoceutics, Dali University, Yunnan Dali 671000, China Dali University Yunnan Dali China

**Keywords:** Asia, goblin spiders, morphology, Orchestininae, taxonomy

## Abstract

Two new species of *Orchestina*, *O.dapojing* Tong & Yang, **sp. nov.** (♂♀) and *O.hyperofrontata* Tong & Yang, **sp. nov.** (♂) are described from Yunnan, China. Descriptions, diagnoses and photographs of habitus and copulatory organs are provided.

## ﻿Introduction

*Orchestina* Simon, 1882 is a species-rich genus of oonopid spider that currently contains 164 extant species (WSC 2024). It has an almost global distribution and occurs in the Northern Hemisphere in the region south of 45°N (Marusik et al. 2018). Currently, 19 species of this genus are known to occur in China (Tong and Li 2011; Liu et al. 2016, 2019; Wang et al. 2021; Lin et al. 2024; Song et al. 2024). Only two species, *O.manicata* Simon, 1893 and *O.striata* Simon, 1909, are known in adjacent Vietnam (WSC 2024).

Although the genus *Orchestina* was well known among arachnologists, there are no global revisions for this genus up to now. The most recent regional revisions on the genus include a description of 18 new species of the Afrotropical region (Henrard and Jocqué 2012) and 85 new species and six known species from the Americas (Izquierdo and Ramírez 2017). Phylogenetic relationships of the species were explored for African species, and two species groups (each with two subgroups) were recognized (Henrard and Jocqué 2012). Species groupings for East Asian or Chinese species have yet to be recognized.

While studying oonopid spiders collected from Cangshan Mountain, Yunnan Province, two new species of the genus *Orchestina* were recognized. It is the first time that this genus has been found in Yunnan. The present paper aims to provide detailed descriptions and illustrations of the two new species, *O.dapojing* Tong & Yang, sp. nov. and *O.hyperofrontata* Tong & Yang, sp. nov.

## ﻿Material and methods

All the specimens used in this study were collected by pitfall trapping and later examined using a Leica M205C stereomicroscope. Details of body parts and measurements were studied under an Olympus BX51 compound microscope. Photos were made with a Canon EOS 750D zoom digital camera (18 megapixels) mounted on the Olympus compound microscope. Endogyne were cleared in lactic acid. All measurements in the text are expressed in millimeters. Terminology and taxonomic descriptions follow Henrard and Jocqué (2012) and Tong and Li (2011). All materials studied are deposited at Shenyang Normal University (**SYNU**) in Shenyang, China.

The following abbreviations are used in the text and figures: **ALE** = anterior lateral eyes; **AUS** = anterior uterine sclerite; **Ex** = dorsolateral extension; **PLE** = posterior lateral eyes; **PME** = posterior median eyes.

## ﻿Taxonomy

### ﻿Family Oonopidae Simon, 1890

#### ﻿Genus Orchestina

Taxon classificationAnimaliaAraneaeOonopidae

Simon, 1882

EB0145DB-C2EA-5310-9E8B-B5CB02FBCF24

##### Type species.

*Schoenobatespavesii* Simon, 1873.

##### Remark.

*Orchestina* is considered a senior synonym of *Ferchestina* Saaristo & Marusik, 2004 (type *F.storozhenkoi* Saaristo & Marusik, 2004) by Platnick et al. (2012: 37).

#### ﻿ Orchestina dapojing

Taxon classificationAnimaliaAraneaeOonopidae

Tong & Yang sp. nov.

5E9332AF-64C5-5DE1-86EE-EA2A1379FA26

https://zoobank.org/B0FF2F6A-2D30-41B5-A664-902C7BD87328

Figs 1 , 2 , 3

##### Type material.

***Holotype*** ♂ (SYNU-764): China, Yunnan Prov., Dali Bai Autonomous Pref., Dali City, Cangshan Mt., Dapojing, pitfall trapping in forest, 25°34′17"N, 100°08′15"E, 2600 m, Z. Yang leg., 25/1/2010; ***Paratypes*** 1 ♀ (SYNU-765), same data as holotype; 1 ♂ (SYNU-766), Cangshan Mt., Jishejing, pitfall trapping in forest, 25°40′29"N, 100°06′36"E, 2600 m, Z. Yang leg., 15/11/2008.

##### Diagnosis.

The new species is similar to *O.apiculata* Liu, Xiao & Xu, 2016 in the shape of the bulb and the long psembolus, but can be distinguished by the palpal tibia narrower than the bulb (Fig. 2A) vs. distinctly wider than the bulb in *O.apiculata* (Liu et al. 2016: fig. 2A, B), triangular labium (Fig. 2D) vs. sub-circular (Liu et al. 2016: figs 1E, 3H), and the tubular anterior uterine sclerite (AUS) (Fig. 3G, H) vs. broad and column-shaped (Liu et al. 2016: fig. 2C, D).

**Figure 1.**
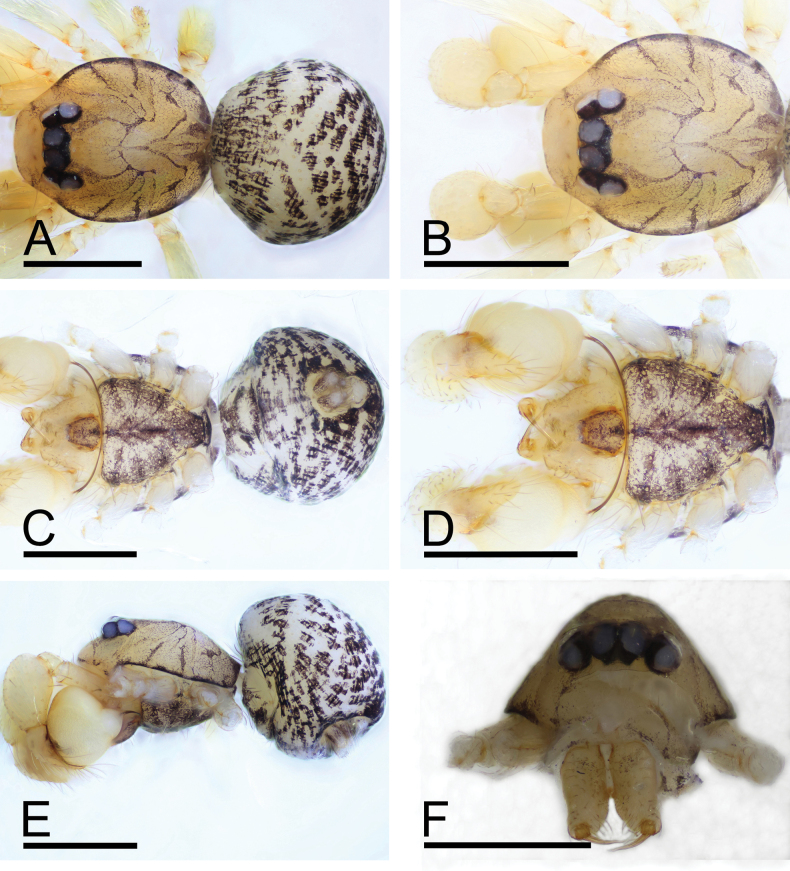
*Orchestinadapojing* sp. nov., male holotype **A, C, E** habitus (dorsal, ventral and lateral views) **B, D, F** prosoma (dorsal, ventral and anterior views). Scale bars: 0.4 mm (**A–F**).

**Figure 2.**
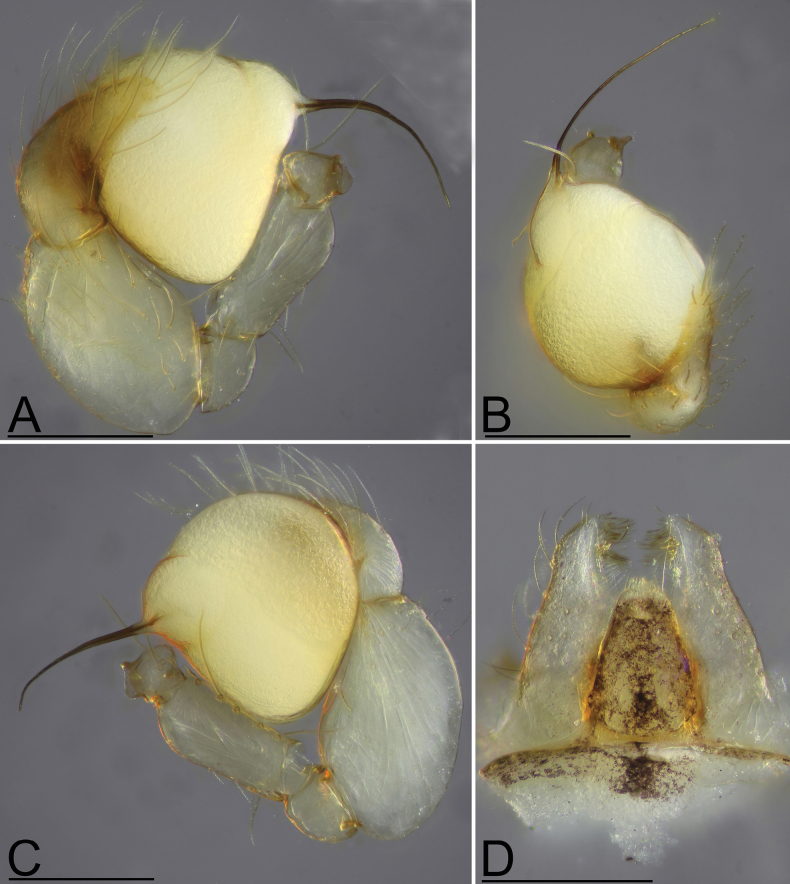
*Orchestinadapojing* sp. nov., male holotype **A–C** left palp (prolateral, dorsal and retrolateral views) **D** endites and labium, ventral view. Scale bars: 0.2 mm (**A–D**).

**Figure 3.**
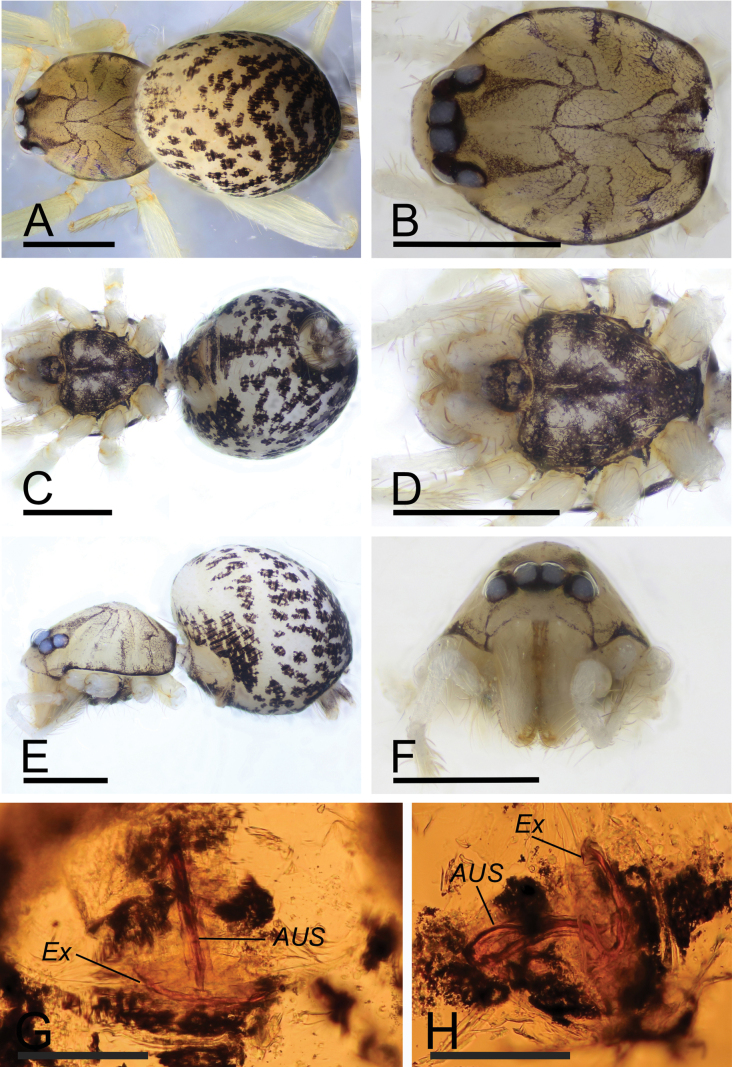
*Orchestinadapojing* sp. nov., female paratype **A, C, E** habitus (dorsal, ventral and lateral views) **B, D, F** prosoma (dorsal, ventral and anterior views) **G** endogyne, dorsal view **H** endogyne, lateral view. Abbreviations: AUS = anterior uterine sclerite; Ex = dorsolateral extension. Scale bars: 0.4 mm (**A–F**); 0.1 mm (**G, H**).

##### Description.

**Male (holotype). *Body***: habitus as in Fig. 1A, C, E; body length 1.33. ***Carapace*** (Fig. 1B): 0.74 long, 0.56 wide; yellow, oval in dorsal view, with net-shaped pattern, pars cephalica slightly elevated in lateral view, with rounded posterolateral corners. ***Eyes*** (Fig. 1B, F): well developed, nearly equal-sized; posterior eye row recurved from above. ***Clypeus*** (Fig. 1B, F): margin unmodified, curved downwards in front view, sloping forward in lateral view, high, ALE separated from edge of carapace by their diameter or more. ***Sternum*** (Fig. 1D): with marginal band and median dark brown patches; setae sparse, needle-like, evenly scattered. ***Mouthparts*** (Figs 1D, F, 2D): chelicerae straight, anterior face unmodified; labium triangular, anterior margin not indented at middle, lateral margins slightly sclerotized, labium pattern darkly spotted but medially delimiting 2 pale, adjacent, oval areas; endites unmodified. ***Abdomen*** (Fig. 1A, C, E): 0.59 long; with gray net-like pattern; pedicel tube short, unmodified. ***Palp*** (Fig. 2A–C): tibia enlarged, length/width ratio = 1.41, more than 2 times as wide as femur; bulb pear-shaped, strongly enlarged, about 1.5 times as wide as tibia; psembolus nearly as long as bulb, whip-like, bent inwards.

**Female (SYNU-765)**: Same as male except as noted. Body: habitus as in Fig. 3A, C, E; body length 1.63. ***Carapace*** (Fig. 3B): 0.74 long, 0.57 wide. ***Abdomen***: 0.89 long. ***Epigaster***: without special external features. ***Endogyne*** (Fig. 3G, H): with medial tubular sclerite (AUS) extending anteriorly, then flipped posteriorly, ending near epigastric furrow; dorsolateral extension (Ex) interrupted anteriorly.

##### Affinities.

The new species is similar to *O.apiculata* and *O.clavigera* Henrard & Jocqué, 2012 (from Kenya). Based on the long, whip-like psembolus, the tubular anterior uterine sclerite (AUS), and the weakly sclerotized endites, the new species should be classified in the *macrofoliata*-subgroup of the *macrofoliata*-group of Henrard and Jocqué (2012).

##### Etymology.

The specific name is a noun in apposition taken from the type locality.

##### Distribution.

Known only from the type locality, Yunnan Province, China.

#### ﻿ Orchestina hyperofrontata

Taxon classificationAnimaliaAraneaeOonopidae

Tong & Yang sp. nov.

E6A3A817-8BEC-58DF-BE88-F4D51BDEAD7D

https://zoobank.org/C9704B06-8A4A-4647-9EF2-ED532C9D2271

Figs 4 , 5

##### Type material.

***Holotype*** ♂ (SYNU-762): China, Yunnan Prov., Dali Bai Autonomous Pref., Dali City, Cangshan Mt., Jishejing, pitfall trapping in forest, 25°40′29"N, 100°06′36"E, 2600 m, Z. Yang leg., 15/11/2008; ***Paratype***: 1 ♂ (SYNU-763), Cangshan Mt., post-fire forest in 1999, pitfall trapping, 25°38′30"N, 100°08′04"E, S. Huang & Y. Zhang leg., 25/4/2009.

##### Diagnosis.

**Figure 4.**
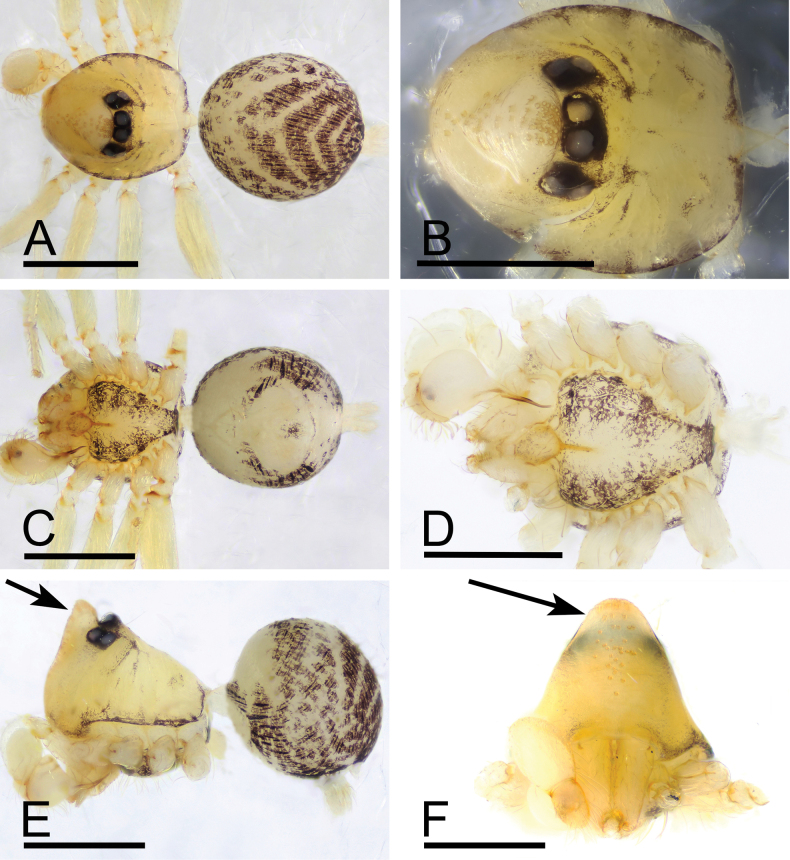
*Orchestinahyperofrontata* sp. nov., male holotype **A, C, E** habitus (dorsal, ventral and lateral views) **B, D, F** prosoma (dorsal, ventral and anterior views), arrows show strongly elevated clypeus. Scale bars: 0.4 mm (**A–F**).

**Figure 5.**
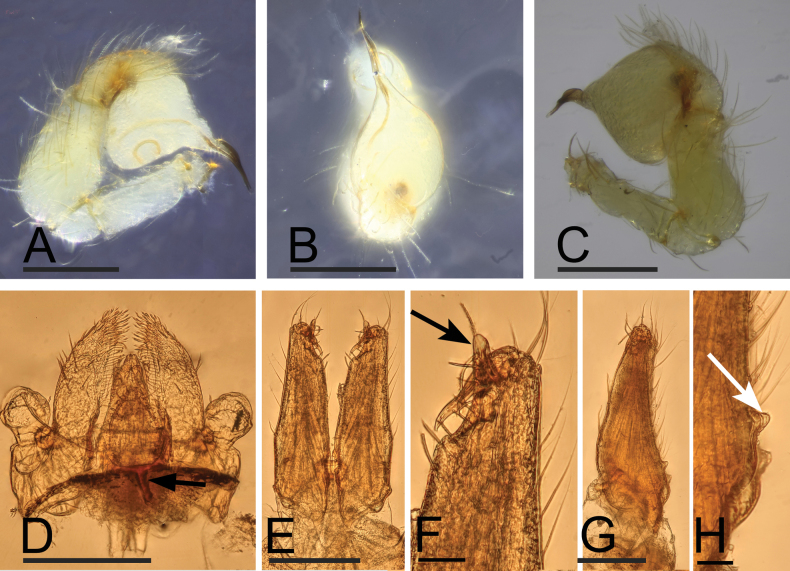
*Orchestinahyperofrontata* sp. nov., male holotype **A–C** left palp (prolateral, dorsal and retrolateral views) **D** endites and labium, ventral view, black arrow shows the Y-shaped sclerotized pattern **E, F** chelicerae, anterior view, black arrow shows the prong **G, H** chelicerae, lateral view, white arrow shows small boss. Scale bars: 0.1 mm (**A–H**).

The new species can be distinguished from all other congeneric species by the strongly elevated clypeus (Fig. 4A, B, E, F) and the prong on the distal part of the chelicerae (Fig. 5F), vs. lacking in congeners.

##### Description.

**Male (holotype). *Body***: habitus as in Fig. 4A, C, E; body length 1.31. ***Carapace*** (Fig. 4B, E): 0.74 long, 0.61 wide; yellow, oval in dorsal view; pars cephalica strongly sloped in lateral view, with rounded posterolateral corners. ***Eyes*** (Fig. 4B): well developed, nearly equal-sized; posterior eye row straight from above; ALE far away from PME. ***Clypeus*** (Fig. 4B, E, F): strongly elevated, curved downwards in front view, sloping forward in lateral view, dorso-apically with hum elevating beyond eyes and provided with dense group of pores. ***Sternum*** (Fig. 4D): with brown patches, anteriorly with a thin median sclerotized bar connected to the labium base (Y-shaped pattern in Fig. 5D). ***Mouthparts*** (Figs 4D, 5D–H): chelicerae basally with small conical boss and distally with well-developed prong partially covering base of fang; labium rounded, lateral and basal margins sclerotized, anterior margin not indented at middle; endites unsclerotized, without serrula. ***Abdomen*** (Fig. 4A, C, E): 0.57 long; with gray net-shaped pattern; pedicel tube short, unmodified. ***Palp*** (Fig. 5A–C): tibia enlarged, length/width ratio = 1.65, about 2 times as wide as femur; bulb globose, about 1.5 times as wide as palpal tibia, seminal duct slightly curved and partly visible through cuticle; psembolus about as long as 1/2 bulb, gradually narrowed.

**Female.** Unknown.

##### Affinities.

The new species *O.hyperofrontata* sp. nov. is similar to *O.utahana* Chamberlin & Ivie, 1935 and *O.obscura* Chamberlin & Ivie, 1942 from the Nearctic in the general shape of the male palp and the presence of prongs on cheliceral promargin (Fig. 5A–C, F; Izquierdo and Ramírez 2017: figs 5B, 15A–C, G–I). Meanwhile, the new species is also similar to *O.kasuku* Henrard & Jocqué, 2012 (from West Africa) in the eye pattern (ALE and PLE not touching PME), the endites without serrula, and the shape of the bulb (Figs 4B, 5A–D; Henrard and Jocqué 2012: figs 378–416). It is reasonable to place the new species *O.hyperofrontata* sp. nov. in the *macrofoliata*-subgroup of the *macrofoliata*-group of Henrard and Jocqué (2012).

##### Etymology.

The specific name is derived from the Greek, *hypero*, meaning beyond, combined with frons, referring to the strongly elevated clypeus.

##### Distribution.

Known only from the type locality, Yunnan Province, China.

## Supplementary Material

XML Treatment for Orchestina

XML Treatment for Orchestina dapojing

XML Treatment for Orchestina hyperofrontata

## ﻿Additional information

### Author ORCIDs

Xiaohan Wang https://orcid.org/0009-0000-4614-9562

Dongju Bian https://orcid.org/0000-0002-9324-8026

Yanfeng Tong https://orcid.org/0000-0002-4348-7029

Zizhong Yang https://orcid.org/0000-0002-2143-8226

### Data availability

All of the data that support the findings of this study are available in the main text.
